# Comparative Genomic Study of Vinyl Chloride Cluster and Description of Novel Species, *Mycolicibacterium vinylchloridicum* sp. nov.

**DOI:** 10.3389/fmicb.2021.767895

**Published:** 2021-12-22

**Authors:** Carlos Cortés-Albayay, Vartul Sangal, Hans-Peter Klenk, Imen Nouioui

**Affiliations:** ^1^Faculty of Science, School of Natural and Environmental Sciences, Newcastle University, Newcastle upon Tyne, United Kingdom; ^2^Faculty of Health and Life Sciences, Northumbria University, Newcastle upon Tyne, United Kingdom; ^3^Leibniz Institute DSMZ – German Collection of Microorganisms and Cell Cultures, Braunschweig, Germany

**Keywords:** bioremediation, polyphasic taxonomy, actinobacteria, bioprospecting, nontuberculous mycobacteria

## Abstract

Advanced physicochemical and chemical absorption methods for chlorinated ethenes are feasible but incur high costs and leave traces of pollutants on the site. Biodegradation of such pollutants by anaerobic or aerobic bacteria is emerging as a potential alternative. Several mycobacteria including *Mycolicibacterium aurum* L1, *Mycolicibacterium chubuense* NBB4, *Mycolicibacterium rhodesiae* JS60, *Mycolicibacterium rhodesiae* NBB3 and *Mycolicibacterium smegmatis* JS623 have previously been described as assimilators of vinyl chloride (VC). In this study, we compared nucleotide sequence of VC cluster and performed a taxogenomic evaluation of these mycobacterial species. The results showed that the complete VC cluster was acquired by horizontal gene transfer and not intrinsic to the genus *Mycobacterium sensu lato*. These results also revealed the presence of an additional *xcb*F1 gene that seems to be involved in Coenzyme M biosynthesis, which is ultimately used in the VC degradation pathway. Furthermore, we suggest for the first time that S/N-Oxide reductase encoding gene was involved in the dissociation of the SsuABC transporters from the organosulfur, which play a crucial role in the Coenzyme M biosynthesis. Based on genomic data, *M. aurum* L1, *M. chubuense* NBB4*, M. rhodesiae* JS60, *M. rhodesiae* NBB3 and *M. smegmatis* JS623 were misclassified and form a novel species within the genus *Mycobacterium sensu lato*. *Mycolicibacterium aurum* L1^T^ (CECT 8761^T^ = DSM 6695^T^) was the subject of polyphasic taxonomic studies and showed ANI and dDDH values of 84.7 and 28.5% with its close phylogenetic neighbour, *M. sphagni* ATCC 33027^T^. Phenotypic, chemotaxonomic and genomic data considering strain L1^T^ (CECT 8761^T^ = DSM 6695^T^) as a type strain of novel species with the proposed name, *Mycolicibacterium vinylchloridicum* sp. nov.

## Introduction

Chlorinated ethenes (CE) are one of the major contaminants of soil and groundwater due to its excessive use in several industries, such as polyvinylchloride industry and plastic manufactory. The physicochemical properties of CE help for their infiltration from water table to the bottom of aquifer. CE stands in the environment longer than other volatile hydrocarbons and has a carcinogenic effect in animal and human health (Henschler, 1994; Volpe et al., 2007). Therefore, a threshold value of chlorinated ethene compounds in drinking water was set up in several countries. CE are presented in three forms, methanes, ethanes and ethenes with tetrachloride (CT), perchloroethene (PCE), trichloroethene (TCE) and vinyl chloride (VC) as common pollutants (Le and Coleman, 2011). The latter is the results of reductive dechlorination of polychlorinated ethenes by anaerobic bacteria (Le and Coleman, 2011). The reduction of VC to ethenes is not an easy step due to the absence and/or inactive microorganisms in the subsurface of ecosystems which led to an increasing rate of VC in groundwater. In addition, this pollutant can be formed naturally in soil after the oxidative degradation of organic matter. For these reasons, VC is considered as a priority contaminant of the groundwater and regulated by the US Environmental Protection Agency (Sass et al., 2005).

The aerobic degradation pathway of VC is the same as ethene where alkene monooxygenase (AKMO), encoded by *etn*ABCD, is the starting point for degrading VC which is transformed to chlorooxirane after AKMO adds O_2_ to its double bond (Hartmans and de Bont, 1992; Jin and Mattes, 2010). The epoxide is associated to coenzyme M (CoM) by epoxyalkane, coenzyme M transferase (EaCoMT) which is encoded by *etn*E, and consequently 2-ketoethyl-CoM is generated (Jin and Mattes, 2010). The latter is transformed to malonate semi aldehyde which is converted to malonate by CoM reductase carboxylase and aldehyde/alcohol dehydrogenase, respectively. CoA-transferase transforms malonate to malonyl-CoA that is converted to acetyl-S-CoA by reductive decarboxylase as the final aerobic VC degradation (de Bont and Harder, 1978). In this regard, two groups of bacteria were identified: (1) VC cometabolizers known for their ability to use ethane as carbon source and found to lack *etn*E gene. These organisms can only degrade ethene to chlorooxiranes while (2) VC assimilators bacteria are able to use VC as sole carbon source and have *etn*ABCD and *etn*E genes (Begley et al., 2012). Moreover, the degradation of VC can also be done through a non-specific oxygenase by ammonia, isoprene, methane, propane and toluene-associated bacteria (e.g. *Methylosinus trichosponum* OB3b and *Nitrosomonas europaea*).

The actinobacterial VC assimilators include strains, such as: *Nocardioides* sp. JS614, *Mycobacterium rhodesiae* JS60 (Coleman et al., 2002), *Mycobacterium rhodesiae* NBB3 (Coleman et al., 2006), *Mycobacterium chubuense* NBB4 (Coleman et al., 2006) and *Mycobacterium smegmatis* JS623 (Coleman and Spain, 2003a). *Mycobacterium aurum* L1^T^ (CECT 8761^T^ = DSM 6695^T^) was the first actinobacteria strain described as a remover of VC from waste gases with high rate of degradation (93%; Hartmans et al., 1985). However, little is known about the ethylene gene cluster for these VC assimilator microorganisms.

*Mycobacterial* strains showed a great adaptability to different contaminated terrestrial and aquatic environments through their ability to produce biosurfactants and to degrade chlorinated pollutants, such as *Mycobacterium chubuense* DSM 44219^T^ and *Mycobacterium obuense* DSM 44075^T^ (Tsukamura et al., 1981; Coleman et al., 2006; Satsuma and Masuda, 2012; Das et al., 2015). These latter strains together with *Mycobacterium aurum* species were transferred to the genus *Mycolicibacterium* defined after the taxonomic revision of the genus *Mycobacterium* based on comparative genomic studies by Gupta et al. (2018). *Mycolicibacterium* genus encompasses fast growing environmental mycobacterial strains of ‘*fortuitum*-*vaccae’* clade which are known for their saprophytic and opportunistic lifestyles and are widely distributed in nature (soil, sediment, water, etc.) including contaminated soils. The genus *Mycolicibacterium* of the family *Mycobacteriaceae* (Chester 1897) housed 88 species with validly published name and with *Mycolicibacterium fortuitum* as the type species.^1^ Like *Mycobacterium sensu lato*, members of this taxon showed yellow or orange and white to cream-coloured colonies. They were characterised by the presence of cell wall rich in lipids and waxes; straight-chain saturated, unsaturated and tuberculostearic (10-methyloctadecanoic) fatty acids and mycolic acid with 60–90 carbon atoms (Magee and Ward, 2012). Genome size from 3.95 to 8.0 Mbp and G + C content between 65.4 and 70.3% (Gupta et al., 2018).

In this present study, *Mycolicibacterium aurum* L1^T^ (CECT 8761^T^ = DSM 6695^T^), potential degrader of VC, was the subject of a comparative genome mapping of ethylene clusters and polyphasic taxonomic studies.

## Materials and Methods

### Strains Cultivation and Maintenance

*Mycolicibacterium aurum* L1^T^ was isolated from a vinyl chloride polluted soil collected at Arnhem, Netherland (Hartmans and de Bont, 1992). The strain was deposited by Dr. Sybe Hartmans (Wageningen Agricultural University, Netherlands) at the German Collection of Microorganisms and Cell Cultures (DSMZ) and the Spanish Type Culture Collection (CECT) under accession numbers DSM 6695^T^ and CECT 8761^T^, respectively. The strain included in this study was obtained from CECT and the strain designation L1^T^ was used in the whole manuscript to avoid confusion. *Mycolicibacterium sphagni* DSM 44076^T^ (obtained from the DSMZ) was found to be the close phylogenetic neighbour of strain L1^T^. These strains were maintained on proteose peptone-meat extract-glycerol agar (PMG; DSMZ 250 medium) at 28°C and conserved as bacterial suspensions in 30%, v/v glycerol at −80°C.

### Cultural and Morphological Characterisation

The cultural properties of strain L1^T^ were evaluated on different agar media: PMG, glucose-yeast extract-malt extract agar (GYM; DSMZ medium 65), International Streptomyces Project (ISP2; Shirling and Gottlieb, 1966), Löwenstein-Jensen medium (LJ; Jensen, 1932), Middlebrook 7H10 agar (MB7H10; Lorian, 1968) and tryptic soy agar (TSA; MacFaddin, 1985) and in presence of at a wide range of temperature 4°C, 10°C, 15°C, 25°C, 28°C, 37°C and 45°C. Anaerobic growth test of strain L1^T^ was examined using an anaerobic bag system (Sigma-Aldrich 68,061).

### Biochemical and Phenotypic Tests

Strain L1^T^ and its phylogenetic neighbour, *M. sphagni* DSM 44076^T^, were examined for a broad range of biochemical tests known to be of value in mycobacterial systematics: arylsulfatase after 3 and 14 days (Tomioka et al., 1990), catalase (de Waard and Robledo, 2007), heat stable catalase (Sequeira de Latini and Barrera, 2008), nitrate reduction (Vincent et al., 2003) and potassium tellurite tolerance (Kilburn et al., 1969; Kent and Kubica, 1985). Moreover, the growth of these strains was also evaluated in the presence of a wide range of carbon, nitrogen substrates and inhibitory compounds using GENIII microplates. The latter were inoculated with a bacterial suspension as described by Nouioui et al. (2017) and then incubated at 28°C for 5 days in an Omnilog device (Biolog Inc., Hayward, United States). The resultants data were analysed using opm package version 1.3 (Vaas et al., 2012, 2013). Furthermore, the enzymatic activities of strains L1^T^ and *M. sphagni* DSM 44076 ^T^ were determined using API coryne kit and following the manufacturer’s instruction (Biomérieux, France). Each test was performed in duplicate.

### Chemotaxonomic Studies

The chemotaxonomic markers relevant to the genus *Mycolicibacterium* were examined for strain L1^T^ and its neighbour *M. sphagni* DSM 44076^T^. Biomass was harvested from cultures, prepared on medium DSMZ 250 and shaked at 250 rpm for 5 days at 28°C. The pellets were washed twice with sterile distilled water and then freeze-dried. Diaminopimelic acid (Schleifer and Schleifer and Kandler, 1972), whole-organism sugars (Lechevalier and Lechevalier, 1970; Staneck and Roberts, 1974) and polar lipids (Minnikin et al., 1984; Kroppenstedt and Goodfellow, 2006) were performed. Cellular fatty acids were extracted following the protocol of Miller (1982) modified by Kuykendall et al. (1988). Gas chromatography (Agilent 6,890 N instrument) was used to analyse the fatty acid methyl esters which were identified using microbial identification (MIDI) system version 4.5 and the MYCO 6 database (Sasser, 1990). Thin-layer chromatographic analyses of mycolic acid extracts of strains L1^T^ and *M. sphagni* DSM 44076^T^ were performed following the protocol of Goodfellow et al. (1976).

### Genome Assembly, Annotation and Comparison

The genomic DNA extraction was performed according to Amaro et al. (2008) and the 16S rRNA gene sequence was generated using the Sanger method (Sanger and Coulson, 1975; Sanger et al., 1977) as a quality control step for the identity of the strain. The genome sequencing was performed on a MiSeq instrument (Illumina) as previously described by Sangal et al. (2015). 300 bp paired-end reads were assembled into contigs using SPAdes 3.9.0 with a k-mer length of 127 (Bankevich et al., 2012). The draft genome sequence was annotated through RAST server (Aziz et al., 2012) and deposited in GenBank database under accession number JACBJQ000000000. The pairwise comparison of average nucleotide identity (ANI) values was performed using the OrthoANIu algorithm and ANI Calculator web tool (Yoon et al., 2017a), at the EzBioCloud portal. Digital DNA–DNA hybridization (dDDH) between the draft genome sequence of strain L1^T^ and their close phylogenetic neighbours were estimated according to the methods described by Meier-Kolthoff et al. (2013).

### Phylogeny

An almost complete 16S rRNA gene sequence (1531 bp, accession number MT478173) was extracted from the draft genome of strain L1^T^ and found to be identical to the sequence obtained by Sanger method. However, the 16S rRNA gene sequence of the nearest neighbours was retrieved from the EzTaxon database (Yoon et al., 2017b). BLAST of the full 16S rRNA gene sequence of isolate L1^T^ was performed against those of validly named species available in EzBioCloud portal (Yoon et al., 2017b). A multiple sequence alignment of all 16S rRNA gene sequences was performed using MUSCLE (Multiple Sequence Comparison by Log- Expectation) algorithm (Edgar, 2004). Phylogenetic trees were constructed using MEGA X software (Kumar et al., 2018) and including Neighbour-Joining (NJ; Saitou and Nei, 1987) and Maximum-Likelihood (ML; Nei and Kumar, 2000) methods with 1,000 bootstrap iterations. The evolutionary distances were calculated using Kimura’s two parameter (Kimura, 1980) and General time reversible (Nei and Kumar, 2000) models.

Phylogenomic tree was inferred from the genome distances calculated with the BLAST distance phylogeny approach (GBDP) using the Type Strain Genome Server pipeline (Meier-Kolthoff and Göker, 2019). The type-based species and subspecies affiliation of strain L1^T^ and the 17 type strains included in the analysis were performed based on the pairwise comparisons mentioned above and according to the thresholds previously reported (Meier-Kolthoff et al., 2013; Yoon et al., 2017a). Tree annotations and visualisations were carried out using the Interactive Tree Of Life (iTOL) webtool (Letunic and Bork, 2021).

### *In silico* Analysis of the *etn* Gene Cluster

The coding sequences comprising the putative gene cluster of ethene (ETH) and vinyl chloride (VC) assimilation pathway were manually mapped and annotated on the draft genome sequence of strain L1^T^ considering criteria of GC reading-frame content (Bibb et al., 1984) and protein domain similarity using ARTEMIS (Berriman and Rutherford, 2003). The ORFs were screened based on their similarity with protein domains of the previously described *etn*EABCD gene cluster of ‘*Mycobacterium smegmatis* JS623’ (accession number: FJ602754.1) and ‘*Mycobacterium chubuense* NBB4’ genome (accession number: NC_018027), which were evidenced after comparison with the Conserved Domains Database (CDD) of NCBI (Marchler-Bauer et al., 2012). The genomic sub-regions of the clusters were compared using BLASTN (Johnson et al., 2008) and visualised with EasyFig 2.2 software (Sullivan et al., 2011).

A sequence similarity network (SSN) of the epoxyalkane coenzyme M transferase (EaCoMT) encoded by the *etn*E gene (Coleman and Spain, 2003b) was constructed to evaluate the taxonomic distribution of the *Mycolicibacterium etn*EABCD gene cluster based on the functional-sequence space of EaCoMT in homologous protein families and its genome context (Copp et al., 2018, 2019). The SSN was generated using EFI-EST (Zallot et al., 2019),^2^ with 1000 homologous proteins from UniProtKB database^3^ and an e-value clustering threshold of 1E-3. The final network was processed and visualised using the organic layout within Cytoscape v. 3.2.0 (Shannon et al., 2003). The genome context of the closest proteins of validly named species was clustered along with the EaCoMT of strain L1^T^. The EaCoMT clusters were preliminary visualised through EFI-GNT^4^ (Zallot et al., 2019) and fully mapped as described above. Only strains with publicly available genome sequences were analysed in this present report. The sequence of EaCoMT of the five mutants, *Mycolicibacterium smegmatis* JS623 (M1-M5) and the partial one of *Mycolicibacterium rhodesiae* JS60 (Coleman and Spain, 2003a,b), was included for the SSN analysis but not considered for further studies.

## Results and Discussion

### Phenotypic Features

Smooth colonies of strain L1^T^ acquired yellow-orange colour, after 5 days of incubation on DSMZ 65 and 250, LJ and MB7H10 media at 28°C and 37°C. Optimal growth was observed on DSMZ 250 medium, pH 7 after 5 days of incubation at 28°C. No colonies were developed under anaerobic condition and neither at 4°C, 15°C, 25°C nor 45°C.

Strain L1^T^ and *M. sphagni* DSM 44076^T^ were unable to reduce nitrate but were able to produce arylsulfatase (after 3 and 14 days) and catalase and reduce potassium tellurite. However, only strain L1^T^ produced a heat stable catalase at 68°C and could be distinguished from its close neighbour by a wide range of metabolic features as shown in Table 1. Strain L1^T^ metabolised D-trehalose and methyl pyruvate (carbon source); butyric acid and citric acid (organic acids); and D-serine and L-arginine (amino acids). It was found to be resistant to nalidixic acid, vancomycin and was able to grow in the presence of guanidine hydrochloride, lithium chloride, up to 4% NaCl and 1% sodium lactate (Table 1).

**Table 1:** Phenotypic features that distinguish strain L1^T^ from *Mycobacterium sphagni* DSM 44076^T^.

	Strain L1^T^	*M. sphagni* DSM 44076^T^
**Carbon source utilisation**
D-galacturonic acid, D-mannose, D-salicin and N-acetyl-D-glucosamine	w	−
D-Trehalose and methyl pyruvate	+	−
**Amino acids**		
D-serine #2 and L-arginine	+	−
Glycine-proline	w	−
**Organic acids**		
Acetoacetic acid, γ-amino-N-butyric acid, and D-malic acid	+	w
Butyric acid and citric acid	+	−
L-lactic acid	w	−
**Inhibitory compounds**		
Guanidine hydrochloride, lithium chloride, 1–4% NaCl and 1% sodium lactate	+	−
**Antibiotics**		
Lincomycin and sodium bromate	w	−
Nalidixic acid	+	w
Vancomycin	+	−
**Biochemical tests (API coryne)**		
Aesculin hydrolysis	−	w
α-glucosidase and pyrrolidonyl arylamidase	w	−

Symbols: −, negative reaction; w, weak reaction; and +, positive reaction. Both strains were able to metabolise D-fructose, D-glucose, D-mannitol, glycerol and myo-inositol (carbon sources); acetic acid, β-hydroxy-butyric acid, bromo-succinic acid, l-malic acid, propionic acid and sodium formate (organic acids); to grow in presence of aztreonam, potassium tellurite, rifamycin sv, tetrazolium blue, tetrazolium violet and Tween 40 (inhibitory compounds); and at pH 6. Both strains showed a positive reaction for alkaline phosphatase (API coryne). In contrast, both strains were unable to oxidise α-D-lactose, β-gentiobiose, β-methyl-D-glucoside, dextrin, D-arabitol, D-cellobiose, D-fucose, D-fructose-6-phosphate, D-galactose, D-glucose-6-phosphate, D-melibiose, D-raffinose, D-saccharic acid, D-sorbitol, D-maltose, gelatin, glucuronamide, inosine, L-fucose, L-rhamnose, N-acetyl-β-D-mannosamine, N-acetyl-D-galactosamine, N-acetyl-neuraminic acid, pectin, stachyose, sucrose, turanose and 3-O-methyl-D-glucose (carbon sources); D-aspartic acid, D-serine #1, L-alanine, L-aspartic acid, L-histidine, L-pyroglutamic acid and L-serine (aminoacids); α-keto-butyric acid, α-keto-glutaric acid, D-glucuronic acid, D-lactic acid methyl ester, hydroxy-butyric acid, L-galactonic acid-γ-lactone, mucic acid, p-hydroxy-phenylacetic acid and quinic acid (organic acids); and were unable to grow in the presence of fusidic acid, minocycline, niaproof, troleandomycin, 8% NaCl and pH 5 (inhibitory compounds). Both strains showed negative enzymatic reactions for β-galactosidase, N-Acetyl-β-glucosaminidase, β-glucuronidase, nitrate reduction, pyrazinamidase, urease and unable to hydrolyze gelatin and metabolise glycogen, ribose and xylose (API coryne).

The chemotaxonomic properties of strain L1^T^ were consistent with its affiliation to the genus *Mycolicibacterium*. Strain L1^T^ showed quantitative and qualitative variations in polar lipid pattern comparing to its close phylogenetic neighbour *M. sphagni* DSM 44076^T^. The major polar lipids for strains L1^T^ and DSM 44076^T^ were diphosphatidylglycerol, phosphatidylethanolamine and phosphatidylinositol (Supplementary Figure S1). The whole-cell hydrolysates of both strains were rich in *meso*-diaminopimelic acid (Supplementary Figure S2), galactose, glucose, mannose and ribose as whole-cell sugars (Supplementary Figure S3). The mycolic acid profiles of strain L1^T^ and *M. sphagni* DSM 44076^T^ contained α-mycolate, methoxymycolate and ketomycolate (Supplementary Figure S4).

The fatty acids patterns of the strain L1^T^ and *M. sphagni* DSM 44076^T^ consisted of C_16:0,_ C_17:1_ ω7c/18 alcohol, C_18:1_ω9c, 10Me-C_18:0_ (tuberculostearic) and 20:0 ALC 18.838/ 20:0 ALC as shown in Supplementary Table S1.

### Phylogenetic and Comparative Genomic

**Figure 1:**
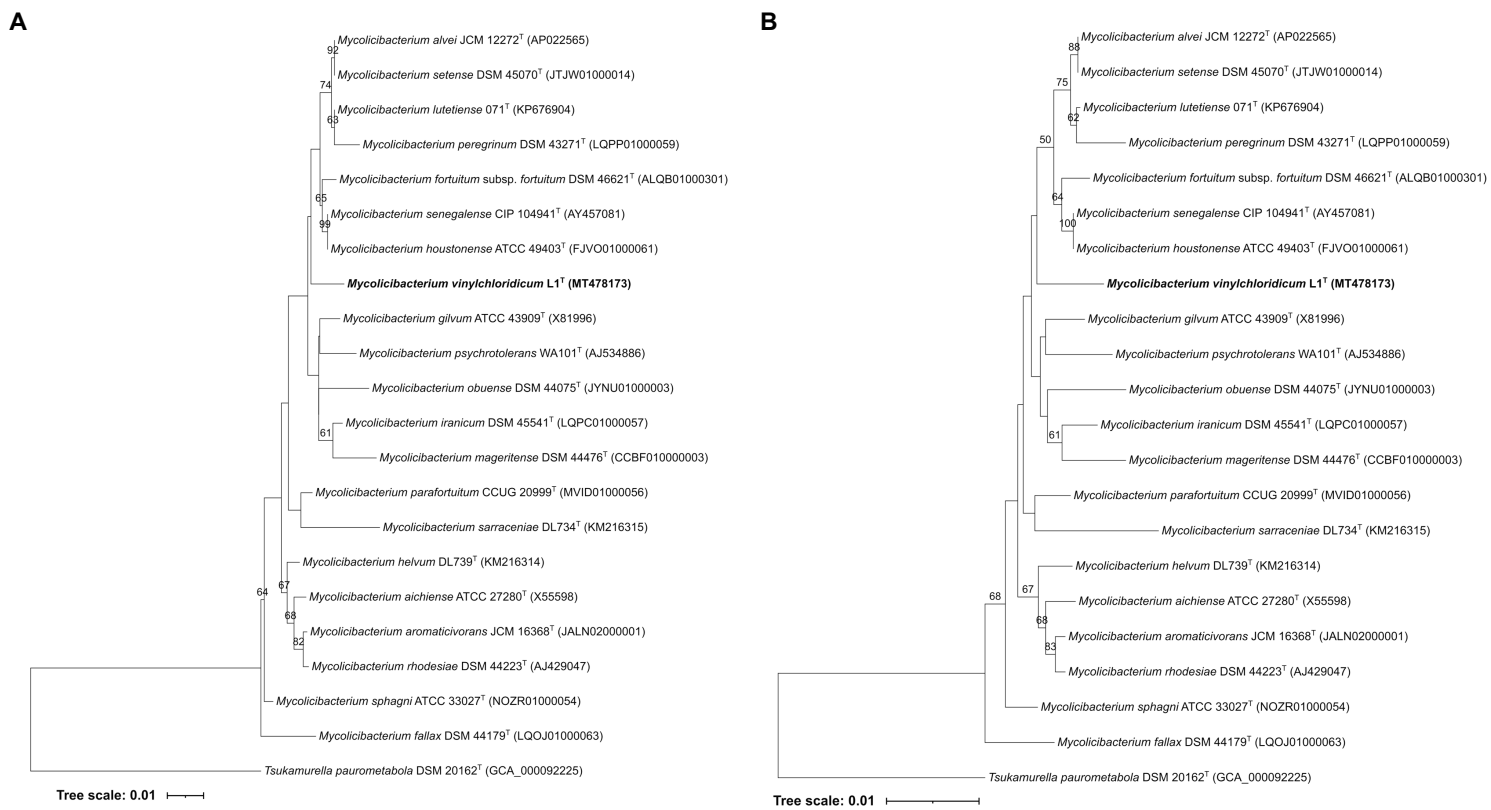
**(A)** Maximum-Likelihood and Neighbour-Joining **(B)** phylogenetic tree based on 16S rDNA gene sequences, showing the taxonomic position of strain L1^T^ within the evolutionary radiation of the genus *Mycolicibacterium*. The numbers above branches are bootstrap support values.

Strain L1^T^ showed 16S rRNA gene sequence (1531pb) similarity values of 98.5% with *M. sphagni* DSM 44076^T^ and 98.8% with *Mycolicibacterium houstonense* ATCC 49403^T^, *Mycolicibacterium senagalense* CIP 104941^T^ and *Mycolicibacterium setense* DSM 45070^T^. These results were not in line with their phylogenetic positions based on the ML and NJ phylogenetic trees (Figures 1A,B). Strain L1^T^ formed a poorly supported distinct branch that is loosely associated to a clade housed the type strains of *Mycolicibacterium alvei, Mycolicibacterium fortuitum* subsp*. fortuitum, M. houstonense, Mycolicibacterium lutetiense, Mycolicibacterium peregrinum, Mycolicibacterium setense* and *M. senagalense* (Figures 1A,B). Hartmans et al. (1985) proposed strain L1^T^ as *Mycobacterium aurum* which was emended as *Mycolicibacterium aurum* (Gupta et al., 2018) as stated above. However, the 16S rRNA gene sequence similarity between strain L1^T^ and the type strain *Mycolicibacterium aurum* DSM 43999^T^ was 97.3% which well below the cut-off point of 98.65% for prokaryotic species demarcation (Kim et al., 2014). Based on this data, strain L1^T^ potentially needs to be defined as a different species.

**Figure 2:**
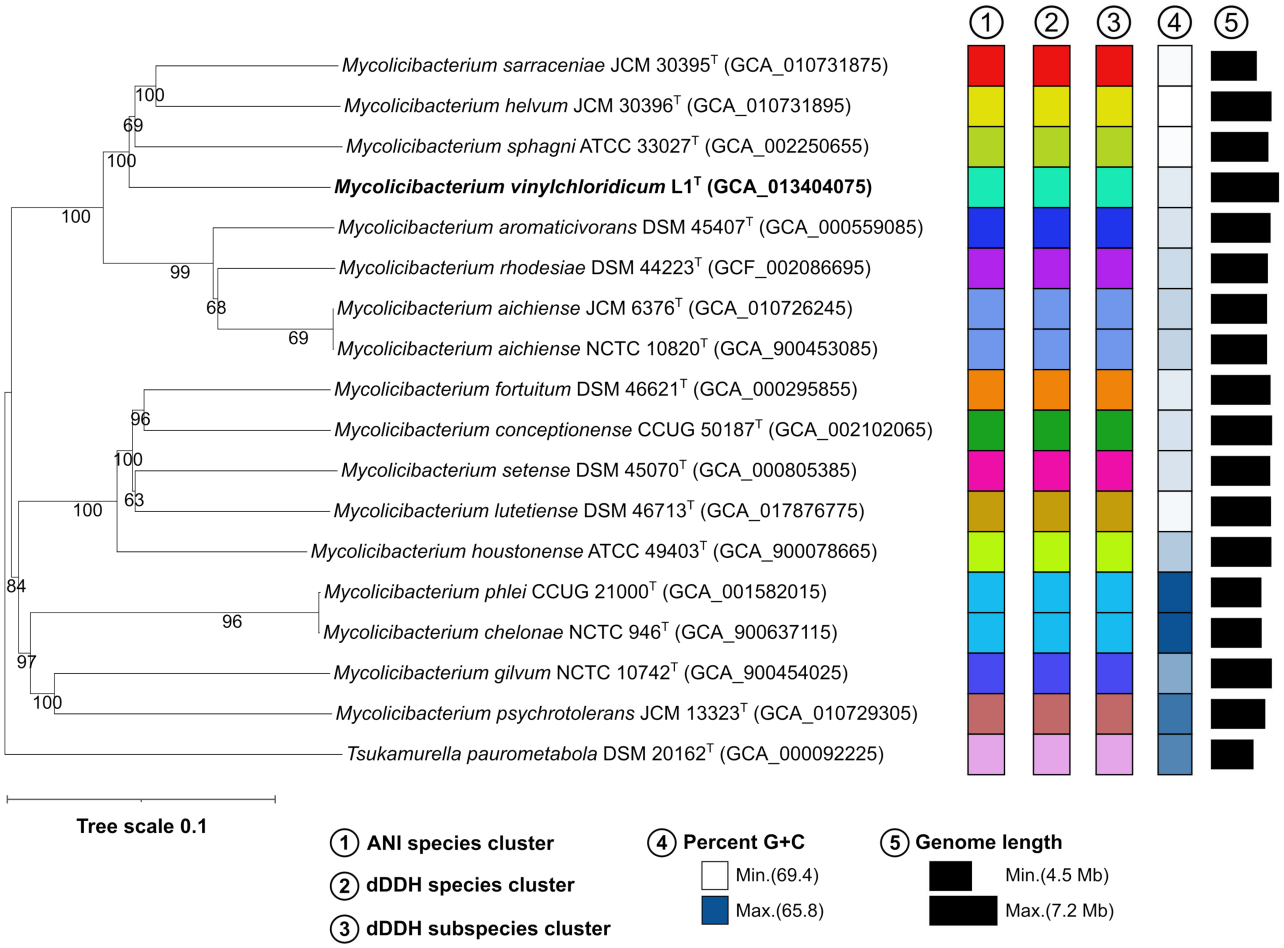
Phylogenomic tree based on GBDP distances calculated from genome sequences, showing the phylogenetic relationship of strain L1^T^ with its close phylogenetic relatives. The numbers above branches are GBDP pseudo-bootstrap support values >60% from 100 iterations, with an average branch support of 89.4%.

In the genome-based phylogeny, strain L1^T^ occupied a well-supported distinct branch closely related to *M. sphagni* ATCC 33027^T^ (GenBank accession number: GCA_002250655) which was next to a subclade housing *Mycolicibacterium sarraceniae* JCM 30395^T^ and *Mycolicibacterium helvum* JCM 30396^T^ (Figure 2). However, *M. senegalense* DSM 43656^T^, *M. houstonenese* ATCC 49403^T^ and *M. setense* DSM 45070^T^ were in distant clade (Figure 2). More confidence can be attributed to the topology of the phylogenomic tree since it is generated from millions of unit characters (Nouioui et al., 2018) and therefore, *M. sphagni* ATCC 33027^T^ is considered as the close neighbour to strain L1^T^.

Strain L1^T^ and *M. sphagni* DSM 44076 ^T^ have genome sizes of 7.1 Mb and 6.0 Mb with 66.6 and 65.9% G + C content, 6,914 and 5,690 coding sequences and 52 and 56 RNAs, respectively. The ANI and dDDH values between the draft genome sequences of strain L1^T^ and its close relative, *M. sphagni* ATCC 33027^T^, were 84.7% and 28.5%, values well below the threshold of 95–96% and 70% used for prokaryotic species delineation, respectively (Wayne et al., 1987; Goris et al., 2007; Richter and Rosselló-Móra, 2009; Lee et al., 2016; Jain et al., 2018).

Since VC degradation trait has been associated with strains belong to *M. aurum*, *M. chubuense*, *M. rhodesiae* and *M. smegmatis* species, the taxonomic affiliation of the reported mycobacterial strains as assimilators of VC was evaluated based on genomic approaches. The dDDH and ANI values between the genome sequence of *M. rhodesiae* NBB3, *M. rhodesiae* JS60, *M. smegmatis* JS623 and *M. chubuense* NBB4 and those of the type strains of their corresponding species were below the defined threshold cited above and confirm that these strains were misclassified and form novel species within the genus *Mycobacterium sensu lato* (Supplementary Table S2). The misclassification of strain JS623 to *M. smegmatis* species was already reported by Garcia and Gola (2016). Therefore, these strains should be referred as *Mycolicibacterium* sp. to avoid confusion and misleading conclusion. In addition, genome mining for VC gene cluster of the reference strains showed that the type strains of *M. aurum*, *M. chubuense, M. rhodesiae* and *M. smegmatis* species devoid from VC gene cluster.

### *In silico* Analysis of the etn Gene Cluster

**Figure 3:**
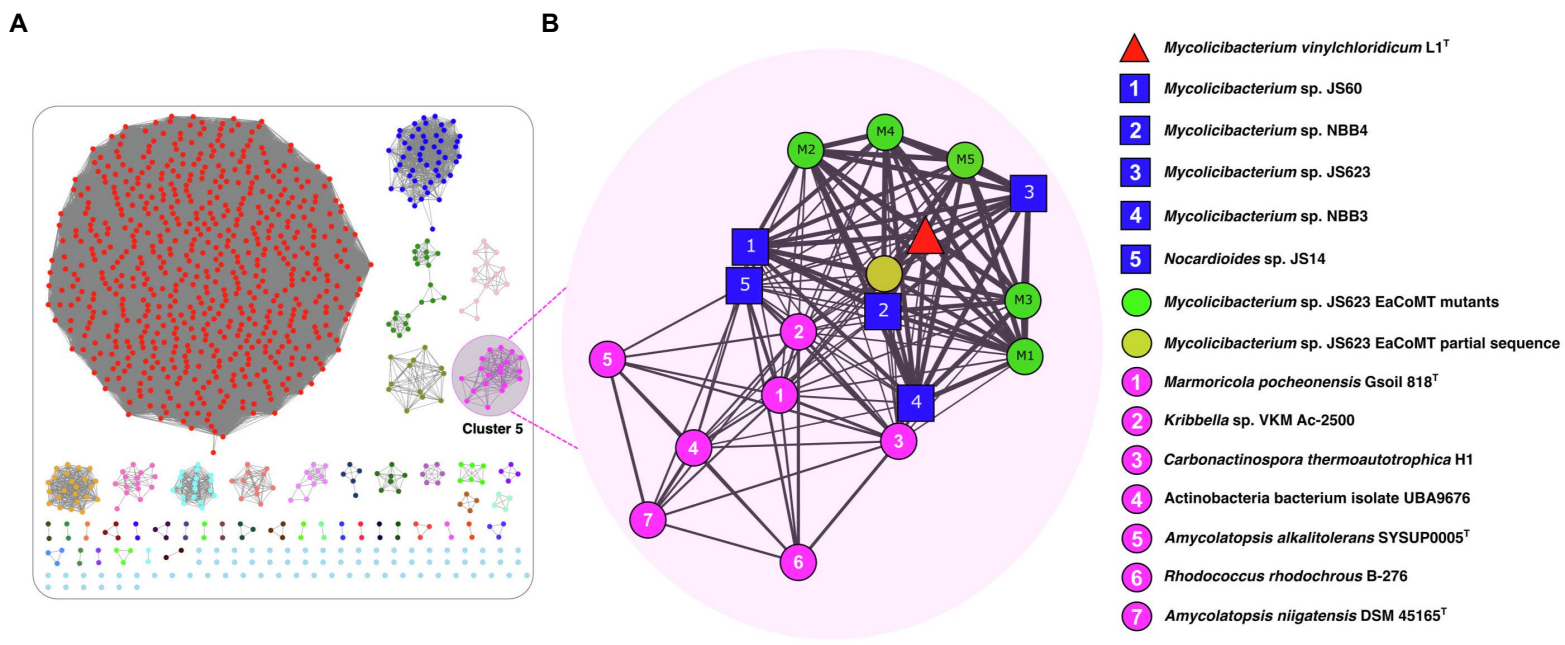
UniProt SSN for the epoxyalkane coenzyme M transferase (EaCoMT) of strain L1^T^. **(A)** Full SSN_170_ with an edge cut-off value of 10^−3^, showing the pairwise sequence similarity relationships among EaCoMT from the strain L1^T^ and their 1,000 closest homologues proteins from UniProtKB database. **(B)** The Cluster 5 (Pink) containing the EaCoMT from the strain L1^T^ (red triangle) and 18 EaCoMTs closest homologues including members of the families *Mycobacteriaceae* and *Nocardioidaceae* (blue squares). Each protein is represented by a circle (node), connected by a line (edge) according to their alignment score and identity reflected by the edge thickness.

The resulting SSN for the EaCoMT (*etn*E) of strain L1^T^ was filtered to include edges with a minimum edge alignment score of 170 (identity >73.5%) and proteins within a minimum and maximum length of 330 and 500 residues (Figure 3). The full SSN_170_ was integrated by 995 protein sequences, segregated into 45 isofunctional clusters and all of them correspond to the catalytic domain of the cobalamin-independent synthase II family (PF01717; Figure 3A). The cluster 5 contained the EaCoMT aminoacidic sequence of strain L1^T^ along with those of *Mycobacteriaceae* and *Nocardioidaceae* (Figure 3B) species, with exception of *Mycolicibacterium moriokaense* GAS496 whose EaCoMT protein sequence showed low similarity with all the other sequences included in the analysis and was subsequently not grouped into any cluster. The sequences incorporated into the cluster 5 belong to strains from the genus *Mycolicibacterium* (6 sequences), *Amycolatopsis* (2 sequences) and *Carbonactinospora, Nocardioides*, *Rhodococcus*, *Kribella* and *Marmoricola* (1 sequence for each); all these taxa belong to the class of *Actinobacteria*.

The EaCoMT sequence of strain L1^T^ showed 98.9, 98.6, 92.6 and 92.3% similarities to those of *M. rhodesiae* JS60, *M. chubuense* NBB4, *M. smegmatis* JS623 and *M. rhodesiae* NBB3, respectively (Figure 3B). However, the sequence identity value decreased to 76.7% between EaCoMT sequence of strain L1^T^ and *Nocardioides* sp. JS614. The latter is known as ETH and VC assimilators (Coleman et al., 2002, 2006; Coleman and Spain, 2003b; Figure 3B).

**Figure 4:**
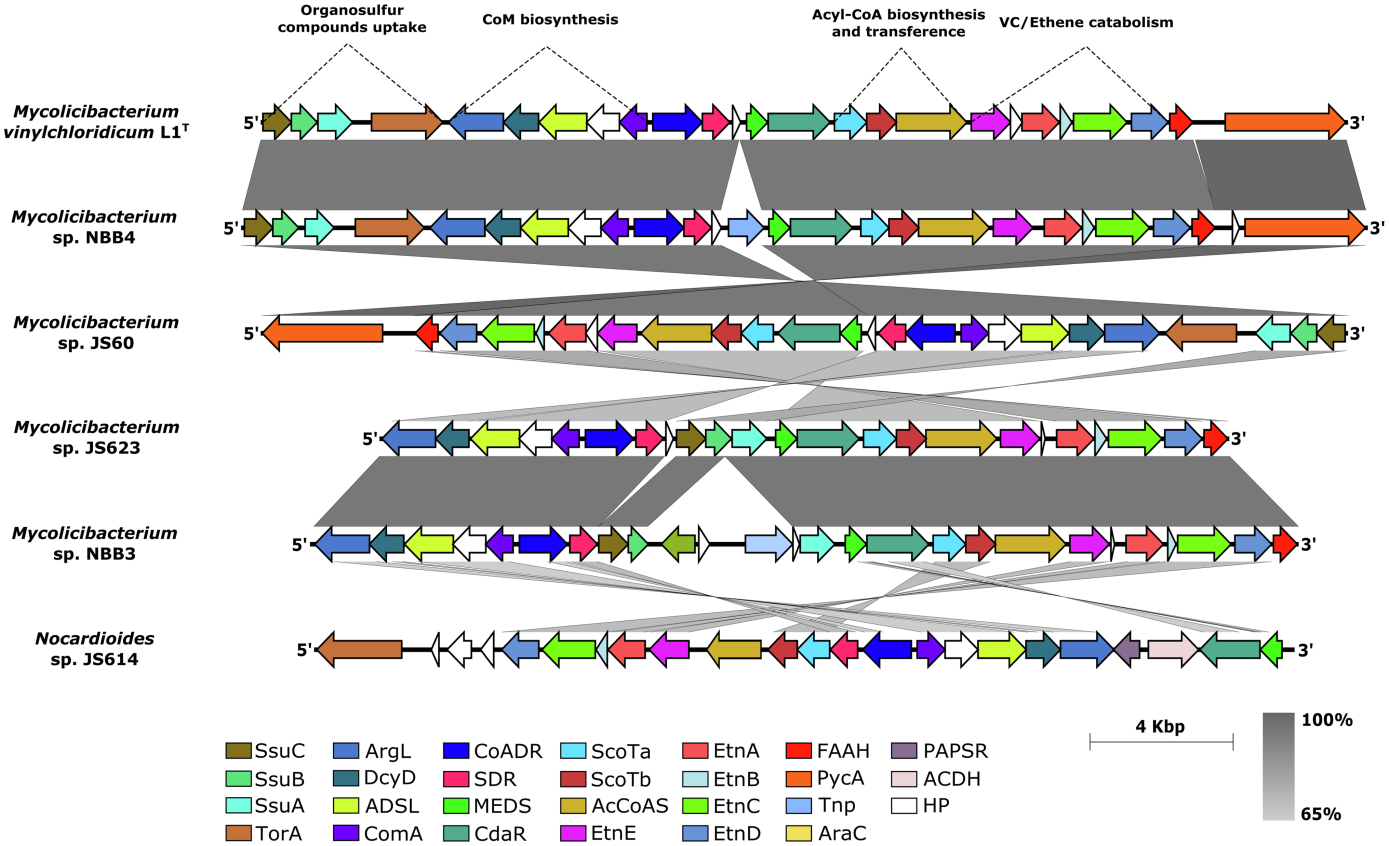
Synteny of the *etn*EABCD cluster sub-region in the genome sequence of strain L1^T^ and five vinyl chloride assimilator strains. SsuB and SsuC: ABC transporter subunits; SsuA: periplasmic aliphatic sulfonate-binding protein; ArgL, argininosuccinate lyase; DcyD, D-cysteine desulfhydrase; AdsL, adenylosuccinate lyase; ComA, phosphosulfolactate synthase; CoADR, CoA-disulphide reductase; SDR, alcohol dehydrogenase; MEDS, methanogen/methylotroph DcmR Sensory domain; CdaR, transcription factor; CoATa and CoATb, two component CoA-transferase; ACoAS, acyl-CoA synthetase; EtnA, monoxygenase β-subunit; EtnB, monoxygenase coupling-effector protein; EtnC, monoxygenase α-subunit; EtnD, alkene reductase; FAAH, fumarylacetoacetate hydrolase; Tnp, transposase; PAPSR, Phosphoadenylyl-sulfate reductase; ACDH, NAD-dependent aldehyde dehydrogenases; and HP, hypothetical proteins.

The genome neighbourhood analysis of the *etn*E gene (EaCoMT) for strain L1^T^ revealed the presence of *etn*ABCD genes, encoding for the protein components of the AkMO (Hartmans et al., 1991), consisting on the monooxygenase β-subunit (EtnA), the monooxygenase coupling-effector protein (EtnB), the monooxygenase α-subunit (EtnC) and the alkene reductase (EtnD; Figure 4). The AkMO complex is responsible for the oxidations of ETH and VC to epoxyethane and chlorooxirane, which are conjugated to CoM yielding 2-hydroxyethyl-CoM and the putative 2-chloro-hydroxyethyl-CoM, respectively. The latter involves in the first steps of the catabolic pathway (Coleman and Spain, 2003a; Mattes et al., 2005). All mycobacterial strains of cluster 5 (Figure 3B) presented the AkMO genes (*etn*ABCD) in their genomes (Figure 4) while the type strains of *M. aurum*, *M. chubuense, M. rhodesiae*, *M. smegmatis* and *M. sphagni* lacked *etn*ACBD cluster.

The Coenzyme M (2-mercaptoethanesulfonic acid), which is necessary for hydroxyalkyl-CoM derivatives generation, is produced by the *xcb*B1,C1,D1,E1 biosynthetic gene cluster (Mattes et al., 2010; Partovi et al., 2018) and was also present in all mycobacterial genomes of cluster 5, and *Nocardioides* sp. JS614, with a slightly different synteny (Figure 4). The gene *xcb*B1 encodes for the phosphosulfolactate synthase (ComA) that catalyses a nucleophilic addition of a sulphite to phosphoenolpyruvate yielding (R)-phosphosulfolactate while argininosuccinate lyase encoded by gene *xcb*C1 is responsible for releasing the phosphate group by mean of β-elimination and generating sulphoacrylic acid. However, the adenylosuccinate lyase encoded by *xcb*D1 was found to presumably catalyse an undetermined co-substrate addition across the sulphoacrylic acid double bond. The *xcb*E1 gene product, D-cysteine desulfhydrase, should be the responsible for the thiolation of the unknown intermediary substrate generated after the XcbD1 reaction, in order to produce the final CoM (Partovi et al., 2018). A new *xcb*F1 gene associated to hypothetical protein of 308 amino acid residues is placed between genes *xcb*B1 and *xcb*E1, showing a strong synteny in all the analysed genomes (Figure 4). This protein found to have a high degree of conservation between the studied strains and an amino acid sequence identity value over 62% (Supplementary Figure S5). Further studies are necessary to determine the function of this protein in the Coenzyme M biosynthesis. Its high conservation within the cluster could be a starting point to decipher the missing intermediaries in the CoM biosynthesis (Partovi et al., 2018).

Two genes encoding for acyl-CoA synthetase (ACoAS) and two component CoA-transferase (CoATa and CoATb; Figure 4) were found in the upstream of gene *etn*E for strain L1^T^. These genes are presumably involved in the generation of Acyl-CoA and the subsequent transference of a CoA group to the malonate in the last steps of Ethene/Vinyl chloride pathway (Mattes et al., 2010).

Following upstream in the genome sequences, strain L1^T^ together with all mycobacterial strains of cluster 5, showed the putative CdaR family transcription factor and the methanogen/methylotroph DcmR Sensory domain (MEDS), which found to be involved in the negative regulation of dichloromethane degradation on methylotrophic bacteria (La Roche and Leisinger, 1991). Both genes are responsible for the transcription of all the catabolic genes downstream including the *etn*EABCD genes (Figure 4).

Furthermore, in the upstream of MEDS regulator, strain L1^T^ housed an alcohol dehydrogenase-associated gene (SDR; Figure 4) which is involved in the dehydrogenation of 2-hydroxyethyl-CoM to 2-ketoethyl-CoM and a CoA-disulphide reductase which could act as a reductive decarboxylase needed to complete the malonyl-CoA assimilation reactions (Mattes et al., 2010). All the analysed strains showed sequence identities above 65% for the conserved *etn*EABCD gene cluster, accessory epoxyalkane catabolic genes (*Sco*Ta and *Sco*Tb), regulators and the genes involved in the coenzyme M biosynthesis.

The *ssu*ABC genes responsible for the uptake of organosulfur compounds, such as sulphate esters, sulfamates, sulfonates and alkanesulfonates during sulphur limited conditions (Beale et al., 2010), were detected approximately 2.7 Kb downstream of CoM biosynthesis genes in the genome of L1^T^. The product of *ssu*A gene corresponds to a periplasmic aliphatic sulfonate-binding protein which binds to the extracellular organosulfur compounds in order to be incorporated by an ABC transporter (proteins SsuB and SsuC) to the cell (Figure 4). An additional gene encoding for S/N-Oxide reductase (T or A; Cheng and Weiner, 2007) was also found next to the *ssu*ABC genes suggesting its participation in the reduction of the uptaked extracellular organosulfur compounds (Figure 4). The sulphate ABC transporter encoding genes (*Ssu*ABC) were present in all the analysed strains except *Nocardioides* sp. JS614.

The boundaries of the described gene cluster showed the presence of genes encoding for transposases, integrases, mobile elements and also flanking direct repeats in most of the studied genomes with exception of *M. smegmatis* JS623 (Figure 4). These genetic elements are known to be associated with genomic islands and lead to further mobilisation, deletion or/and insertion of a complete genomic region (Schmidt and Hensel, 2004; Juhas et al., 2009; da Silva Filho et al., 2018). These findings indicate that these mycobacterial studied strains have acquired the VC degrader feature *via* horizontal gene transfer in order to adapt and survive in the environment.

### Description of *Mycolicibacterium vinylchloridicum* sp. nov.

*M. vinylchloridicum* (vi.nyl.chlo.ri’di.*cum*. N.L. neut. n. *vinylchloridicum*, vinyl chloride; N.L. neut. Adj. *vinylchloridicum*, related to vinyl chloride).

Aerobic, fast growing actinobacterium that develops colonies with yellow-orange colour, after 5 days of incubation on DSMZ 65 and 250, LJ and MB7H10 media. Optimal growth is observed after 3 days of incubation at 28°C on DSMZ 250 medium, pH 7. It is able to metabolise D-fructose, D-glucose, D-mannitol, glycerol and myo- inositol, D-trehalose (carbon source); acetic acid, butyric acid, β-hydroxy-butyric acid, citric acid, bromo-succinic acid, l-malic acid, propionic acid, sodium formate and methyl pyruvate (organic acids); and D-serine and L-arginine (amino acids). It is resistant to nalidixic acid, rifamycin sv, vancomycin and able to grow in the presence of aztreonam, guanidine hydrochloride, lithium chloride, 1% sodium lactate and tetrazolium blue, tetrazolium violet, and up to 4% (w/v) NaCl. It produces alkaline phosphatase, arylsulfatase after 3 and 14 days, catalase and heat stable catalase and reduce potassium tellurite. Whole-cell hydrolysates are rich in *meso*-diaminopimelic acid and galactose, glucose, mannose and ribose as cell sugars. The polar lipid pattern of strain L1^T^ contains diphosphatidylglycerol, phosphatidylethanolamine, phosphatidylinositol, phosphoglycolipid, unidentified glycolipids and unknown phosphoaminolipid. The mycolic acid profile of strain L1^T^ contains α-mycolate, methoxymycolate and ketomycolate. The major fatty acids (>10%) consist of C_16:0,_ C_18:1_ ω9c, C_17:1_ ω 7c/18 alcohol and 10Me-C_18:0_. The G + C content is 66.6 mol% and the genome size is 7.1 Mbp.

The type strain L1^T^ (DSM 6695^T^ = CECT 8761^T^) was isolated from vinyl chloride polluted soil, collected at Arnhem, Netherland. The GenBank accession number of the 16S rRNA gene is MT478173. The Whole-Genome Shotgun project has been deposited at DDBJ/ENA/GenBank under the accession JACBJQ000000000. The version described in this paper is version JACBJQ010000000.

## Overview and Significance

Improvements of the systematics of these mycobacterial VC assimilator strains and their assignment to the corresponding species rank are crucial for their prospective roles in bioremediation. *M. aurum* L1^T^, an actinobacteria degrader of VC, could be distinguished from its close neighbour, *M. sphagni* DSM 44076^T^, by its phenotypic and genomic features and therefore, it merits to be affiliated to a novel species with the proposed name *Mycolicibacterium vinylchloridicum* sp. nov. Genome comparison based on dDDH and ANI showed that *M. rhodesiae* NBB3, *M. rhodesiae* JS60, *M. smegmatis* JS623 and *M. chubuense* NBB4 were misclassified and are new candidate species within the genus *Mycolicibacterium* (Supplementary Table S2). The genome sequence of the type strains of *M. aurum* NCTC 10437^T^, *M. chubuense* DSM 44219^T^*, M. rhodesiae* DSM 44223^T^, *M. smegmatis* NCTC 8159^T^ and *M. sphagni* ATCC 33027^T^ devoid from VC gene cluster. This present report clarifies which mycobacterial species have the VC degrader feature and highlights the importance in attaching a species name to a strain that has potential application in bioremediation as example.

Comparative genomic mapping and analyses showed that the complete VC gene cluster of strain L1^T^, *M. chubuense* NBB4 and *M. rhodesiae* JS60 was acquired by lateral gene transfer and it is not intrinsic to the mycobacterial taxa. These findings are in line with its absence in the genome of other *Mycolicibacterium* strains, such as its close neighbour. The comparative analyses of the coenzyme M biosynthetic gene cluster of the studied strains highlighted the presence of a well conserved hypothetical protein-associated gene between *xcb*B1 and *xcb*E1 genes. The detected gene could be a starting point for further molecular studies to determine its function and decipher the remaining intermediary substrates for the CoM biosynthesis. Moreover, the conserved genomic position of the S/N-Oxide reductase encoding gene (*tor*A) next to the *ssu*ABC gene cluster in all the *Mycobacterium* genomes questions the role of torA related to the uptaken organosulfur by ssuABC proteins. Therefore, we propose that *tor*A gene product involves in the dissociation of the organosulfur from the *ssu*ABC transporters and consequently, it can be used by cells for the Coenzyme M biosynthesis. These data can be used for further molecular and biochemical research studies to decipher all the remaining steps in the VC degradation pathways.

## Data Availability Statement

The datasets presented in this study can be found in online repositories. The names of the repository/repositories and accession number(s) can be found in the article/Supplementary Material.

## Author Contributions

IN: conceptualization, writing—original draft preparation, supervision, and project administration. IN, CC-A, VS, and H-PK: methodology, validation, and writing—review and editing. IN, CC-A, and VS: software and data curation. IN, CC-A, VS and H-PK: formal analysis and visualisation. IN, CC-A, and H-PK: investigation. IN and H-PK: resources. All authors have read and agreed to the published version of the manuscript.

## Conflict of Interest

The authors declare that the research was conducted in the absence of any commercial or financial relationships that could be construed as a potential conflict of interest.

## Publisher’s Note

All claims expressed in this article are solely those of the authors and do not necessarily represent those of their affiliated organizations, or those of the publisher, the editors and the reviewers. Any product that may be evaluated in this article, or claim that may be made by its manufacturer, is not guaranteed or endorsed by the publisher.

## Footnotes

^1^ https://lpsn.dsmz.de/genus/mycolicibacterium

^2^ http://efi.igb.illinois.edu/efi-est/

^3^ https://www.uniprot.org/update

^4^ http://efi.igb.illinois.edu/efi-gnt/
