# Supplementary material for: Comparative Genomic Study of Vinyl Chloride Cluster and Description of Novel Species, Mycolicibacterium vinylchloridicum sp. nov
Source: Front Microbiol. 2021 Dec 22;12:767895. doi: 10.3389/fmicb.2021.767895 (PMC8727900; doi:10.3389/fmicb.2021.767895)
Supplement: Supplementary file 1 [file Presentation_1.pdf]

Supplementary Material

Comparative genomic study of vinyl chloride cluster and description of novel *s*pecies*,* *Mycolicibacterium vinylchloridicum* sp. nov.

Carlos Cortés-Albayay[1], Vartul Sangal[2], Hans-Peter Klenk[1], Imen Nouioui[3*]

[1] School of Natural and Environmental Sciences, Faculty of Science, Newcastle University, Newcastle upon Tyne, United Kingdom.

[2] Faculty of Health and Life Sciences, Northumbria University, Newcastle upon Tyne, United Kingdom.

[3] Leibniz Institute DSMZ – German Collection of Microorganisms and Cell Cultures, Braunschweig, Germany.

*** Correspondence:**

Imen Nouioui

imen.nouioui@dsmz.de

Supplementary Table 1. Fatty acids profiles of strain L1^T and the type strain of *Mycolicibacterium sphagni*, its closest phylogenomic neighbour.

**Fatty acids profiles (%)**	**Strain L1^T**	***Mycolicibacterium sphagni* DSM 44076^T**
$C_{14:0}$	8.6	9.1
$C_{16:1}$ ω6c	6.7	6.3
$C_{16:0}$	24.3	23.5
Sum in feature 2<br>$C_{17:1}$ ω7c/ 18 alcohol	16.9	21.8
$C_{18:1}$ ω9c	14.6	9.0
10Me-$C_{18:0}$	11.6	12.0
Sum in feature 3<br>20:0 ALC 18.838/ 20:0 ALC	4.5	7.3
$C_{10:0}$	1.2	1.0
$C_{12:0}$	2.4	1.7
$C_{16:1}$ ω7c	4.0	2.5
$C_{18:0}$	1.2	2.9
$C_{20:0}$	-	1.2
Summed Feature 1<br>8-Me-$C_{16.0}$/10-Me-$C_{16:0}$	3.2	1

Only fatty acids ≥ 1% are considered. -, fatty acid is absent.

Supplementary Table 2. dDDH and ANI pairwise comparisons between the genome sequences of all the mycolicibacterial strains analysed in this study.

			dDDH percentage											
	**Assembly accession**		**1**	**2**	**3**	**4**	**5**	**6**	**7**	**8**	**9**	**10**	**11**	**12**
Mycolicibacterium sp. L1^{T}	GCA_013404075.1	**1**	-	28.5	20.0	30.0	29.9	27.0	60.3	19.8	20.1	19.9	20.2	20.3
Mycolicibacterium sphagni ATCC 33027^{T}	GCA_002250655.1	**2**	28.5	-	19.5	29.5	29.6	26.4	28.6	19.6	19.8	19.7	19.9	20.0
Mycolicibacterium aurum NCTC 10437^{T}	GCA_900637195.1	**3**	20.0	19.5	-	19.6	19.6	19.7	19.8	20.1	20.1	20.2	23.0	22.7
Mycolicibacterium helvum JCM 30396^{T}	GCA_010731895.1	**4**	30.0	29.5	19.6	-	32.5	26.7	30.2	19.3	19.9	19.9	19.8	20.0
Mycolicibacterium sarraceniae JCM 30395^{T}	GCA_010731875.1	**5**	29.9	29.6	19.6	32.5	-	26.3	30.0	19.6	20.1	19.8	20.0	20.0
Mycolicibacterium rhodesiae DSM 44223^{T}	GCA_002086695.1	**6**	27.0	26.4	19.7	26.7	26.3	-	26.9	20.0	19.8	19.8	20.1	20.1
Mycobacterium rhodesiae JS60	GCA_000230935.2	**7**	60.3	28.6	19.8	30.2	30.0	26.9	-	19.7	19.9	19.8	20.1	20.4
Mycobacterium rhodesiae NBB3	GCA_000230895.3	**8**	19.8	19.6	20.1	19.3	19.6	20.0	19.7	-	20.1	21.4	20.5	20.7
Mycolicibacterium smegmatis NCTC 8159^{T}	GCA_001457595.1	**9**	20.1	19.8	20.1	19.9	20.1	19.8	19.9	20.1	-	20.3	20.8	20.9
Mycobacterium smegmatis JS623	GCA_000328565.1	**10**	19.9	19.7	20.2	19.9	19.8	19.8	19.8	21.4	20.3	-	20.8	20.7
Mycobacterium chubuense NBB4	GCA_000266905.1	**11**	20.2	19.9	23.0	19.8	20.0	20.1	20.1	20.5	20.8	20.8	-	24.7
Mycolicibacterium chubuense DSM 44219^{T}	GCA_001044255.1	**12**	20.3	20.0	22.7	20.0	20.0	20.1	20.4	20.7	20.9	20.7	24.7	-

			ANI percentage											
	**Assembly accession**		**1**	**2**	**3**	**4**	**5**	**6**	**7**	**8**	**9**	**10**	**11**	**12**
Mycolicibacterium sp. L1^{T}	GCA_013404075.1	**1**	-	84.7	75.9	85.7	85.3	84.0	94.9	75.7	76.2	75.9	76.9	76.7
Mycolicibacterium sphagni ATCC 33027^{T}	GCA_002250655.1	**2**	84.7	-	75.5	85.3	85.4	83.2	84.6	75.3	75.6	75.7	76.3	76.5
Mycolicibacterium aurum NCTC 10437^{T}	GCA_900637195.1	**3**	75.9	75.5	-	75.5	75.9	75.8	75.7	76.6	76.8	76.8	80.4	80.1
Mycolicibacterium helvum JCM 30396^{T}	GCA_010731895.1	**4**	85.7	85.3	75.5	-	86.7	83.4	85.5	75.3	75.7	75.7	76.0	76.2
Mycolicibacterium sarraceniae JCM 30395^{T}	GCA_010731875.1	**5**	85.3	85.4	75.9	86.7	-	83.1	85.4	75.5	76.2	75.8	76.2	76.5
Mycolicibacterium rhodesiae DSM 44223^{T}	GCA_002086695.1	**6**	84.0	83.2	75.8	83.4	83.1	-	83.8	75.5	76.1	76.0	76.4	76.7
Mycobacterium rhodesiae JS60	GCA_000230935.2	**7**	94.9	84.6	75.7	85.5	85.4	83.8	-	75.5	76.2	75.9	76.7	76.7
Mycobacterium rhodesiae NBB3	GCA_000230895.3	**8**	75.7	75.3	76.6	75.3	75.5	75.5	75.5	-	76.5	78.2	77.7	77.2
Mycolicibacterium smegmatis NCTC 8159^{T}	GCA_001457595.1	**9**	76.2	75.6	76.8	75.7	76.2	76.1	76.2	76.5	-	77.0	77.4	77.6
Mycobacterium smegmatis JS623	GCA_000328565.1	**10**	75.9	75.7	76.8	75.7	75.8	76.0	75.9	78.2	77.0	-	77.6	77.5
Mycobacterium chubuense NBB4	GCA_000266905.1	**11**	76.9	76.3	80.4	76.0	76.2	76.4	76.7	77.7	77.4	77.6	-	81.7

Mycolicibacterium chubuense DSM 44219^T	GCA_001044255.1	**12**	76.7	76.5	80.1	76.2	76.5	76.7	76.7	77.2	77.6	77.5	81.7	-

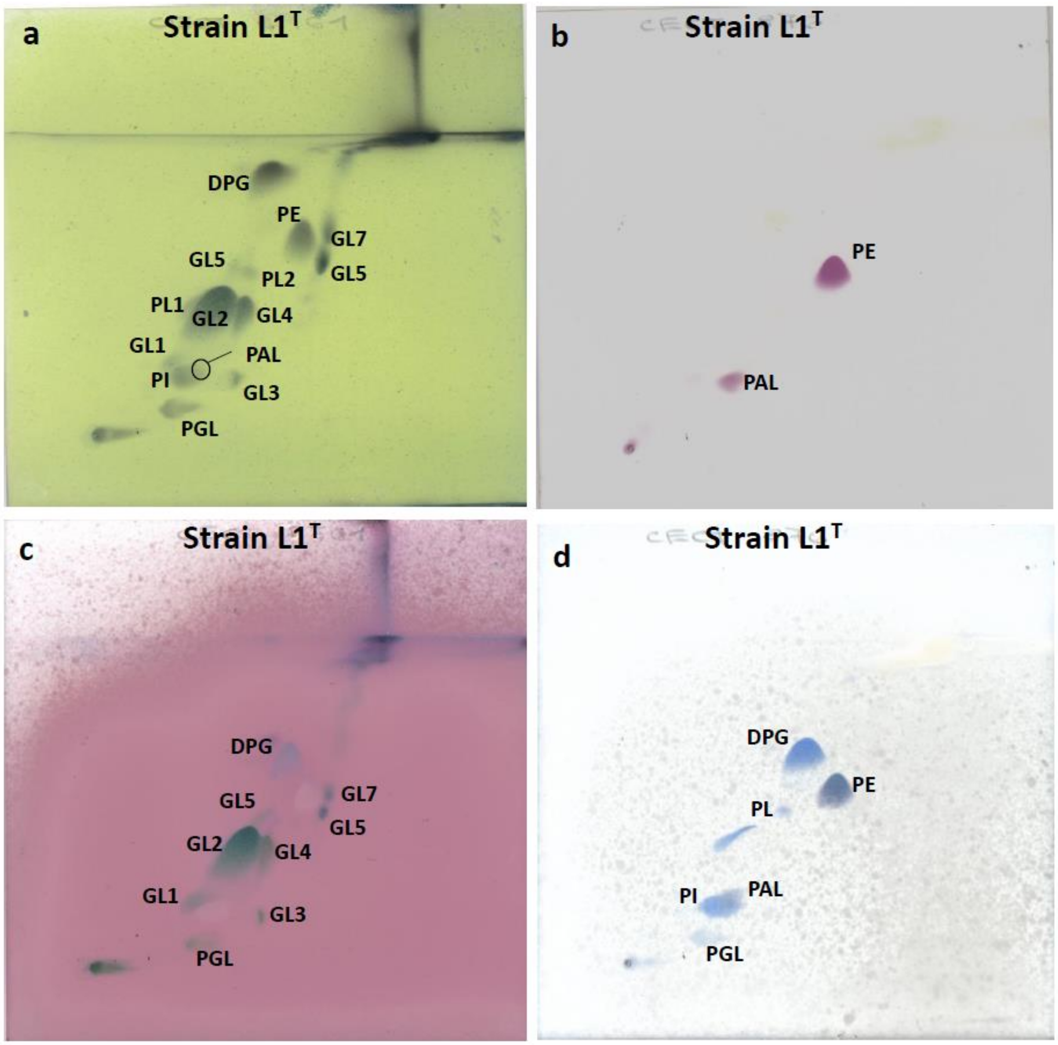

Supplementary Figure 1. Two-dimensional TLC plates of polar lipids extracted from strain L1^T stained with molybdatophosphoric acid (a), ninhydrin (b), anisaldehyde (c) and molybdenum blue (d). Key: DPG, diphosphatidylglycerol; PE, phosphatidylethanolamine; PI, phosphatidylinositol; PGL, phosphoglycolipid; PAL, phosphoaminoplipid, GL, glycolipid, L, lipids and PL, phospholipids. Solvent1: chloroform: methanol: distilled water (65:25:4 v/v/v/); solvent 2: chloroform: glacial acetic acid: methanol: distilled water (80:12:15:4 v/v/v).

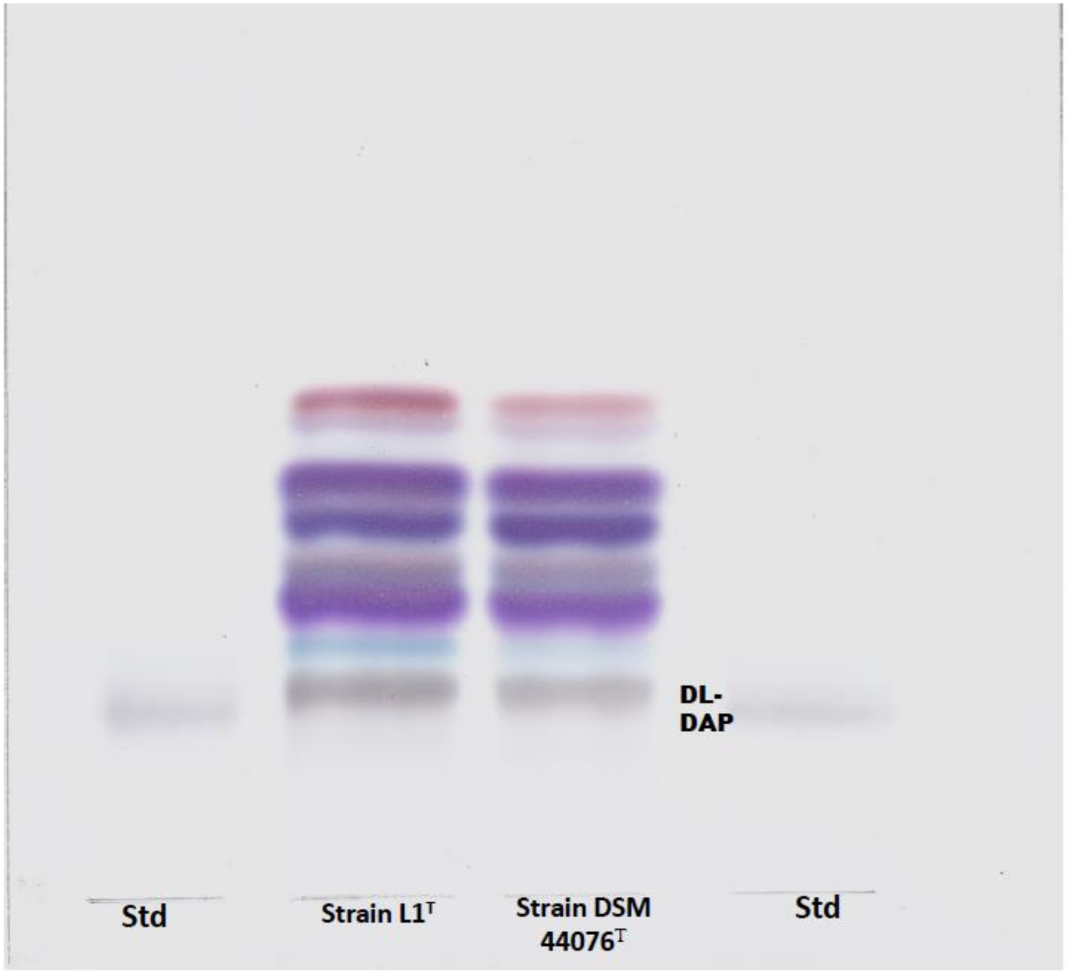

Supplementary Figure 2. Thin layer chromatography of a separation of A2pm isomers from whole cell hydrolysates.

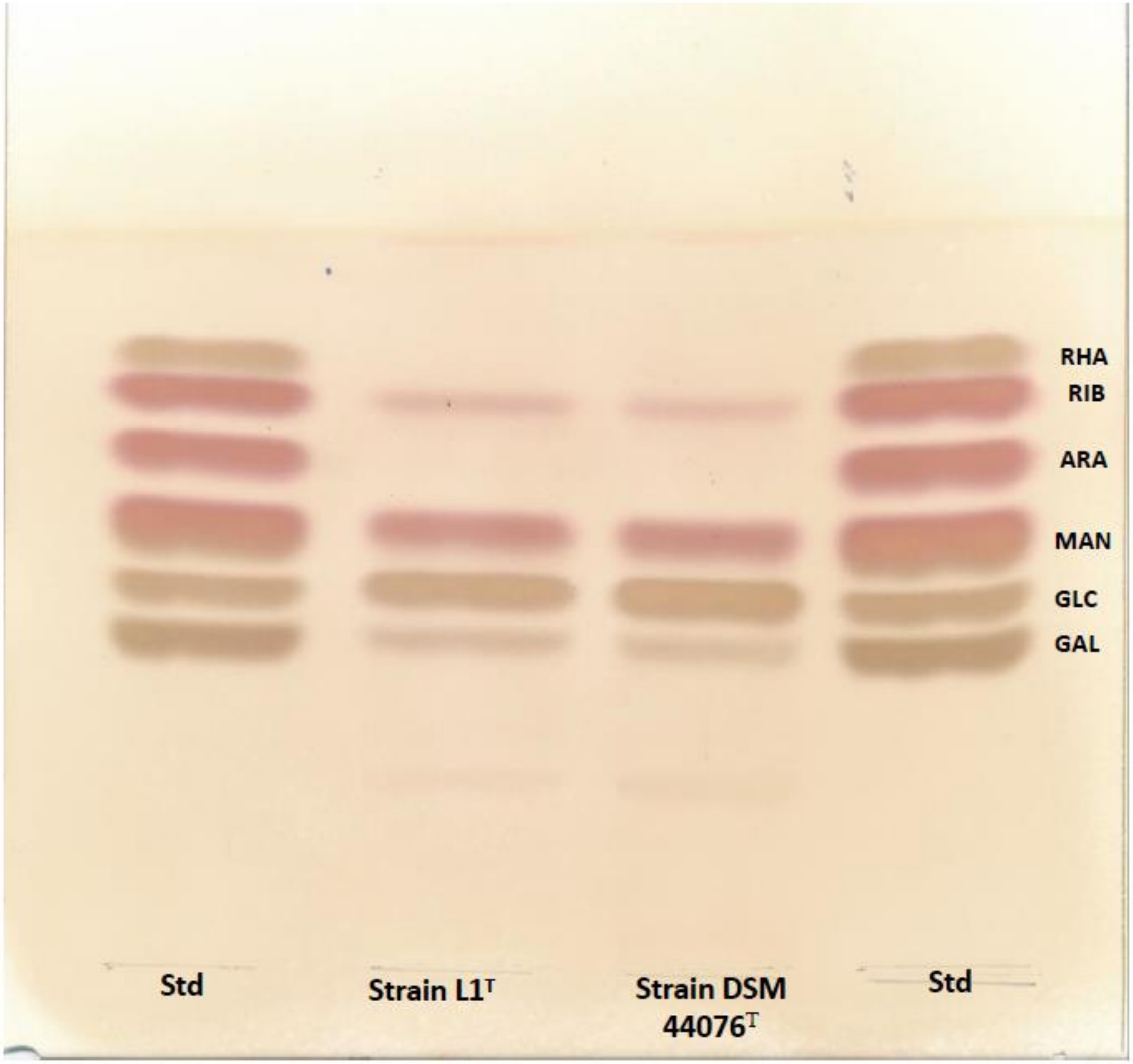

Supplementary Figure 3. Thin layer chromatography of whole cell sugars (RHA, rhamnose; RIB, ribose; ARA, arabinose; MAN, mannose; GLC, glucose; GAL, galactose).

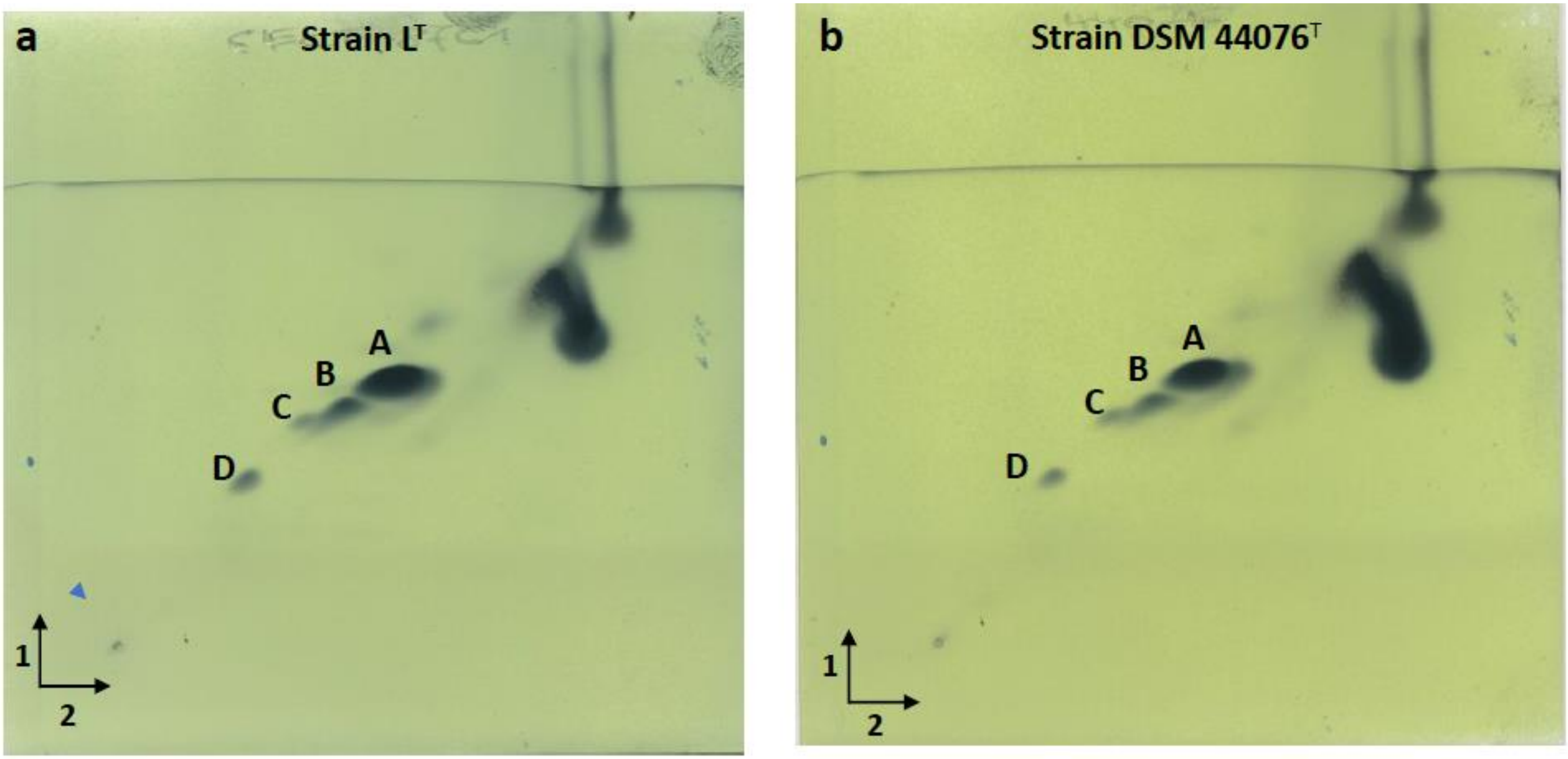

Supplementary Figure 4. Two-dimensional DC plates of mycolic acids extracted from strain L1^T (a) and *M. sphagni* DSM 44076^T(b) stained with molybdatophosphoric acid (SigmaP1518). A, α-mycolate; B, methoxymycolate; C, ketomycolate, D, unknown lipid.

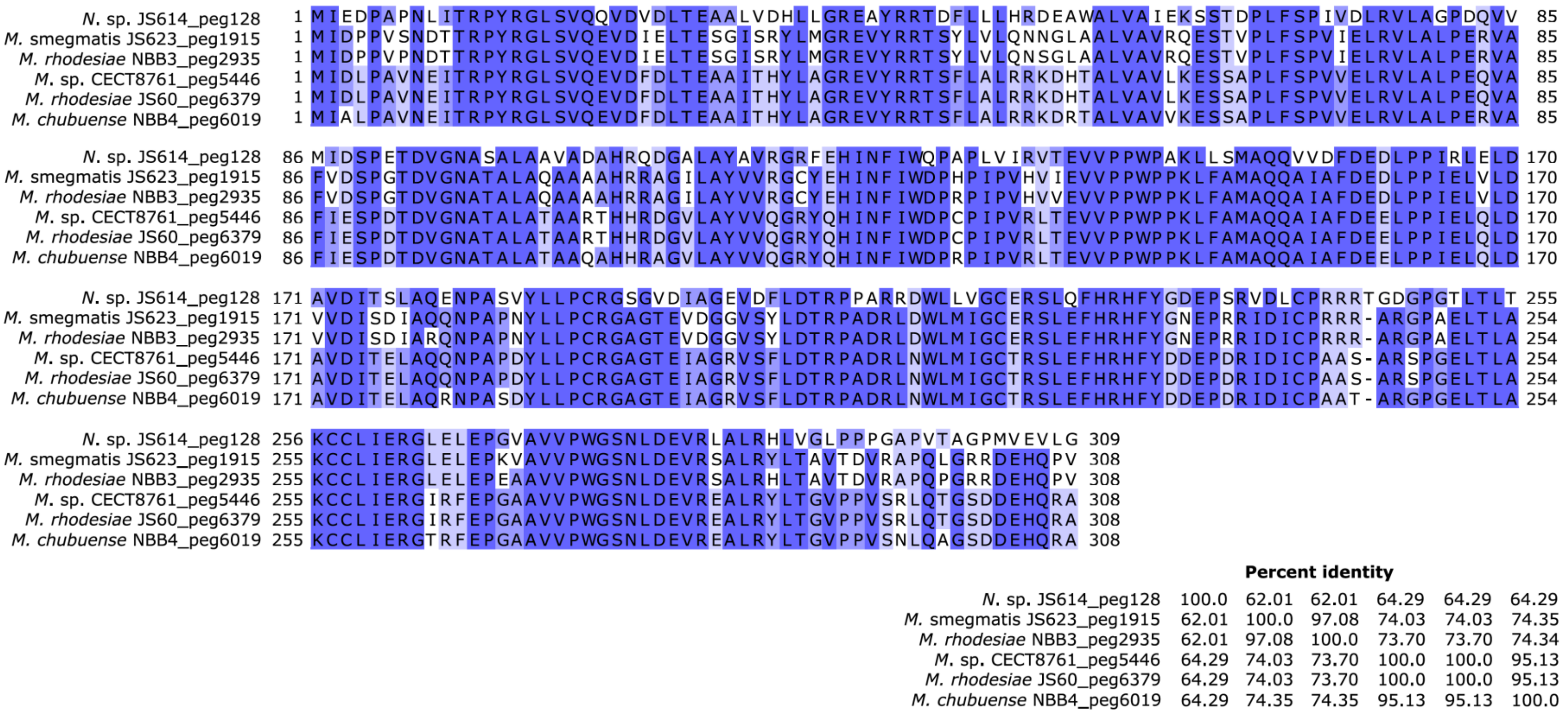

Supplementary Figure 5. Multiple sequence alignment of a new hypothetical protein encoding gene found in the coenzyme M biosynthetic gene cluster (*xcb*B1C1D1E1) of all the *Mycolicibacterium* genome sequences. The hypothetical protein found in the coenzyme M biosynthetic gene cluster of L^{T} and all its relatives genome sequences, showed a minimum identity percentage of 62.01%. The multiple sequence alignment was performed using the Clustal Omega (1.2.4) webtool with the default parameters.
